# Synthesis of heterocycle based carboxymethyl cellulose conjugates as novel anticancer agents targeting HCT116, MCF7, PC3 and A549 cells

**DOI:** 10.1038/s41598-025-14146-1

**Published:** 2025-08-09

**Authors:** Reham A. Mohamed-Ezzat, Zeinab A. Elshahid, Shaimaa A. Gouhar, Sawsan Dacrory

**Affiliations:** 1 Chemistry of Natural and Microbial Products Department, Pharmaceutical and Drug Industries Research Institute, National Research Centre, Cairo, Egypt; 2 Medical Biochemistry Department, Medical Research and Clinical Studies Institute, National Research Centre, Cairo, Egypt; 3 Cellulose and Paper Department, National Research Centre, Cairo, Egypt

**Keywords:** Synthesis, Pyridine, Anticancer, Carboxymethyl cellulose, Cancer, Chemistry, Materials science

## Abstract

Toward developing anticancer agents, heterocycle-based carboxymethyl cellulose conjugates have been synthesized. 2-Cyano-*N*′-(aryl/heteroarylethylidene)acetohydrazides and ethyl 2-cyano-3-(heteryl)acrylates were utilized as precursors for the synthesis of pyridine-based compounds. The chemical structures of the synthesized derivatives were characterized using various spectroscopic techniques, including ^1^H-, ^13^C-NMR, Fourier transform infrared spectroscopy (FTIR), as well as scanning electron microscopy (SEM).The anticancer effects of compounds on HCT-116, MCF-7, PC3 and A549 cancer cell lines were investigated and their cytotoxicity against RPE-1 normal cells was estimated to determine their safety. Compounds **4b** and **7c** exhibit high selectivity toward cancer cells while maintaining a strong safety margin for normal cells. The results demonstrated that the novel heterocycle-based carboxymethyl cellulose conjugates are promising and can be further evaluated as a potential therapeutic agent.

**Supplementary Information:**

The online version contains supplementary material available at 10.1038/s41598-025-14146-1.

## Introduction

Cancer is a complex and heterogeneous disease characterized by abnormal, uncontrolled cell division, leading to tumor formation and the invasion of surrounding healthy tissues. It is the second leading cause of death globally, following cardiovascular diseases, accounting for one in every six deaths worldwide. This alarming prevalence highlights the urgent need to develop more effective therapeutic agents to combat the disease^1^.

**Fig. 1:**
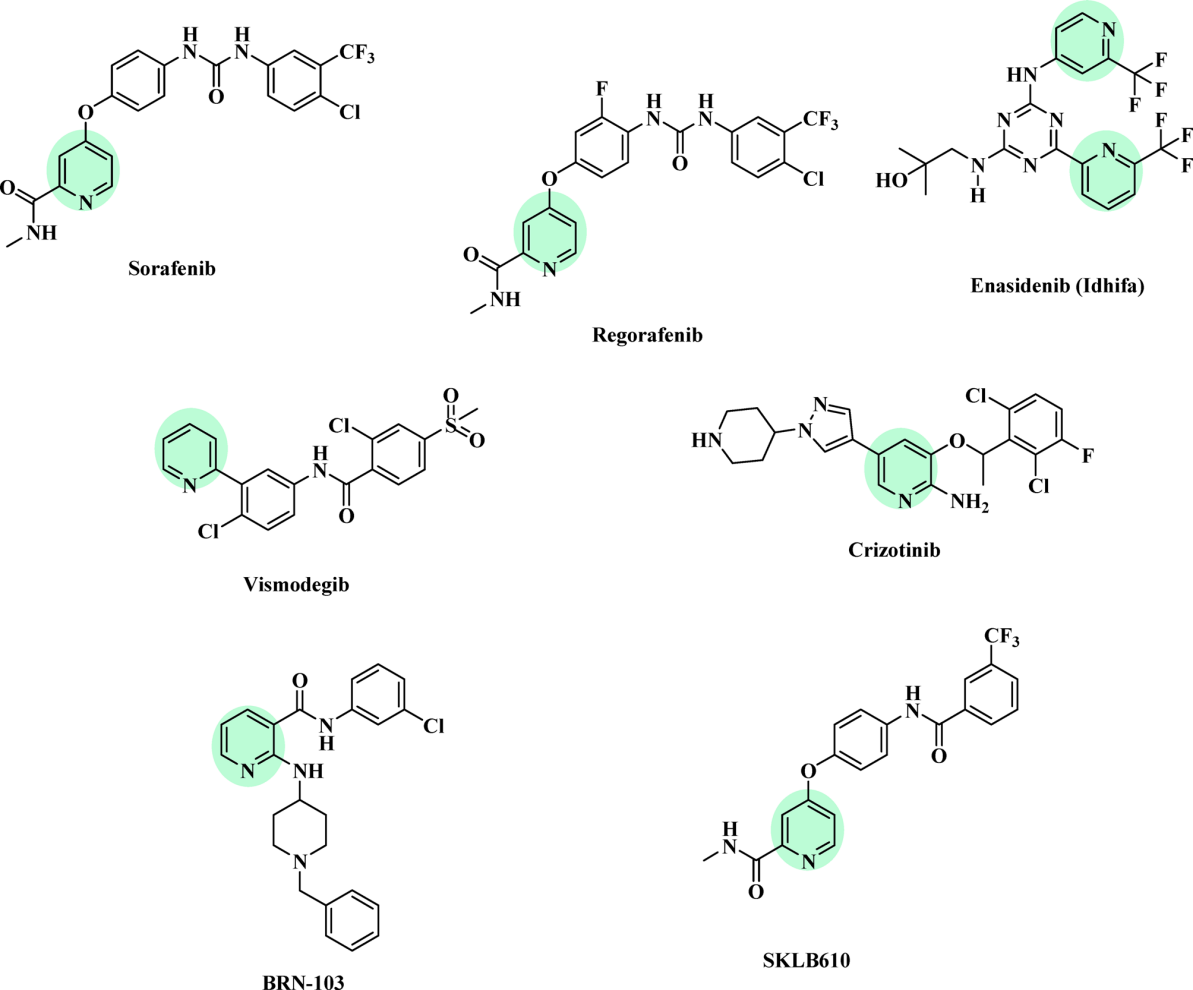
Targeted anticancer agents containing pyridine scaffolds.

Due to its significant impact on quality of life, cancer remains a major global health concern. The rising incidence of cancer, along with the resistance of cancer cells to existing treatments, has intensified the search for new anticancer drugs with enhanced potency, selectivity, improved pharmacokinetics, and reduced toxicity^2^. Over decades of extensive research, many anticancer agents have been developed as structural analogs of pyrimidines, purines, hormones, vitamins, amino acids, and other essential metabolites^3^. Among these, oxygen- and nitrogen-containing heterocyclic compounds have demonstrated promising therapeutic potential against various diseases, including cancer^1^. Heterocyclic-based anticancer drugs exhibit significant antineoplastic activity. Both pyridine and pyrimidine are naturally occurring in biological systems, including genetic material. These core structures play crucial roles in various biological processes and cancer pathogenesis, making them attractive scaffolds for the development of novel therapeutic agents^2^. Heterocyclic compounds have a profound and wide-ranging impact on medicinal chemistry and the pharmaceutical industry. They serve as key components in the expanding field of antimetabolites. Their critical importance has led to extensive use in treating severe diseases, including cancer and malignancies^4^. Nitrogen-containing heterocyclic derivatives, such as azines and pyridine frameworks, are essential in both chemistry and biology (Fig. 1). They are widely utilized in pharmaceuticals, vitamins, natural products, and the synthesis of functional reagents^5–8^. A novel and efficient method for synthesizing various types of pyrimidines has been developed and reported^9–17^. Pyridines have also been explored as potential anticancer agents^18,19^. Heterocyclic compounds containing the pyridine nucleus exhibit a diverse range of biological activities, including anticancer, antioxidant, antimicrobial, and antiviral properties. These compounds have demonstrated significant therapeutic and biological potential across multiple applications^20,21^.

On the other side, biopolymers are indispensable materials for the chemical industry, finding many applications due to their biocompatibility, biodegradability, and low toxicity^22,23^. Among these biopolymers, cellulose and its derivatives have great concern. It is a renewable, cost- effective and abundant material^24^.

Carboxymethyl cellulose (CMC) is a wildly used cellulose derivative; it is a water soluble polymer with carboxmethyl group that attached to some of the hydroxyl groups of the glucopyranose monomers in the cellulose backbone. CMC participate in various applications water treatment^25^, drug delivery^26^ and food packaging^27,28^. Herein, in this work CMC heterocyclic composite (ECFA, ABOC) has prepared and investigated via ^1^H-, ^13^C-NMR, FTIR, SEM, XRD and TGA.

## Result and discussion

### Synthesis

**Fig. 2:**
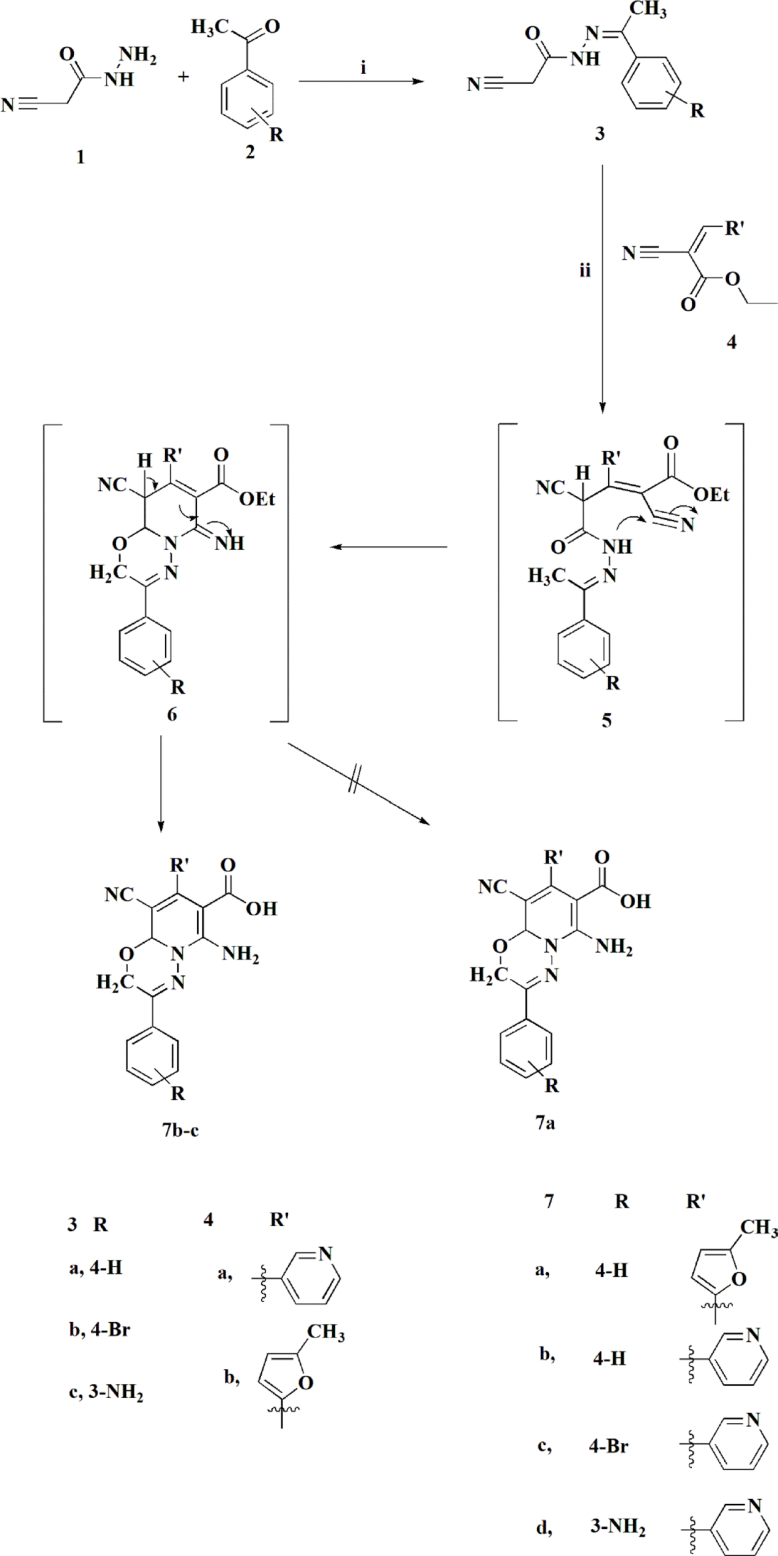
Synthetic pathway for compounds 7**a-d**. Reagents and conditions: **i)** EtOH, reflux, 1 hour. **ii**) AcOH/MeOH (2:1), reflux, 3h.

New derivatives of pyrido[2,1-*b*][1,3,4] oxadiazine-7-carboxylic acid (**7**) were synthesized as depicted in figure 2. This approach in synthesizing of those novel derivatives is a continuation of our recent program of synthesizing the pyridooxadiazine ring^29^. The approach to access compound **7** was achieved starting from synthesizing the ethylidene-2-cyanoacetohydrazide derivatives (**3**) through reacting the cyano acid hydrazide **(1**) with the acetophenone derivatives (**2**) under reflux in ethanol for one hour^29^. Further reacting the substituted ethylidene-2-cyanoacetohydrazides **3** with the ethyl 2-cyano-3-(pyridin-3-yl)acrylate (**4**) yielded the substituted pyrido[2,1-*b*][1,3,4] oxadiazine-7-carboxylic acid (**7**).

**Fig. 3:**
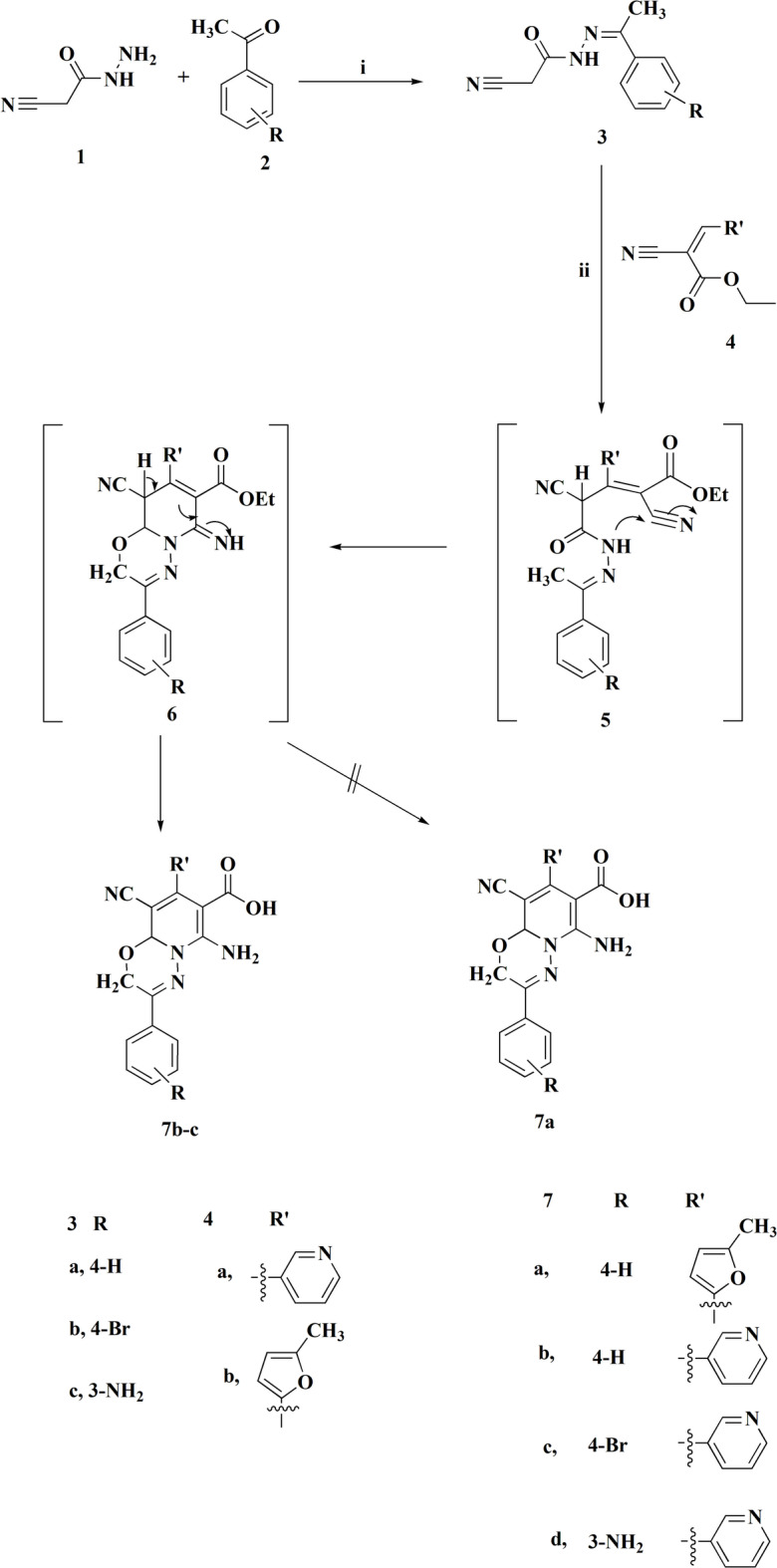
Suggested modifications of CMC with the synthesized compounds 4b & 7c.

The proposed synthesis mechanism of compound **7** likely follows a Michael addition, where the active methylene group of compound **3** reacts with the double bond in compound **4** to form the intermediate 5. Its worthy to note that the starting compounds **3** was synthesized according to previous procedures^19^. The resulting intermediate **5** then undergoes cyclization to form compound **6**. Subsequent hydrolysis of the latter compound in the presence of acetic acid afforded compound **7**. The assignment of the chemical structures of compounds **7** was detected on the basis of its spectral data and elemental analysis. As an example, ^1^H NMR spectrum of the compound **7c** showed two singlets at δ 4.47 & 4.96 ppm, respectively, which are corresponding to the methylene and CH protons accordingly. Signals between δ 7.36 to 8.70 ppm were observed for the eight aromatic protons on the two substituted benzene rings. Additionally, singlet signal for the amino and hydroxyl groups appeared at δ 11.00 ppm. Herein, compound **7b** & **7c** were synthesized under identical conditions, whereas compound **7a** was not generated under the same reaction condition. The derivative **7d** is previously synthesized^29^; in the present study its biological evaluation against HCT116, MCF7, PC3 and A549 cells is reported. Furthermore, the preparation of the pyridine-based carboxymethyl cellulose conjugates is accomplished (Fig. 3). Additionally, ethyl 2-cyano-3-(5-methylfuran-2-yl)acrylate **4b** was reacted with compound **8** to yield the corresponding furan-based carboxymethyl cellulose conjugates.

### FTIR- analysis

**Fig. 4:**
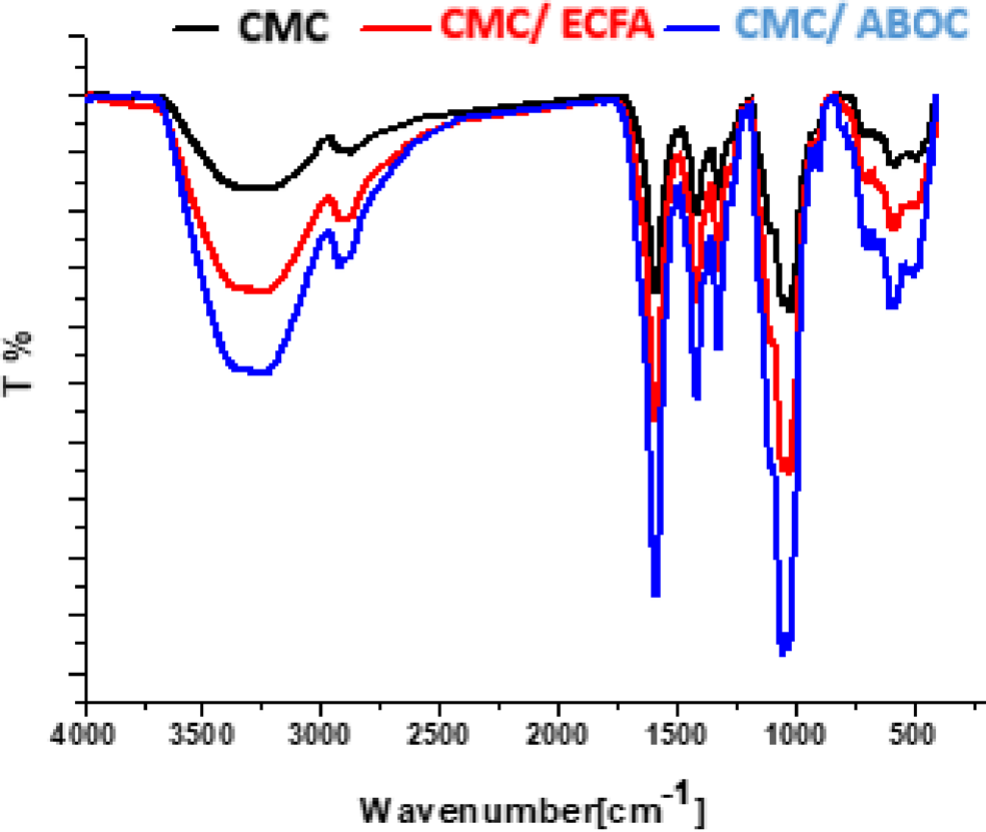
FTIR of CMC and composites.

FTIR is the most useful tool to describe the compounds function group. Figure 4 shows FTIR of CMC and CMC with heterocyclic compounds (**ECFA** & **ABOC**). CMC spectra shows different peaks at 3500 cm^−1^, 2900 cm^−1^, 1650 cm^−1^, and 1040 cm^−1^ at 550 cm^−1^ corresponding to OH stretching, C-H stretching, C = O of carboxylic group, ether linkage of glucose unite and C-Br stretching^30,31^. Spectra of CMC composite through CMC interaction with heterocyclic compounds (ECFA & ABOC show that the proceeded peaks of CMC of OH, CH, C = O, and C-O- C became more intensity and sharp. It may be returned to the homogeneity between the compounds^32,33^.

### Scanning electron microscope

**Fig. 5:**
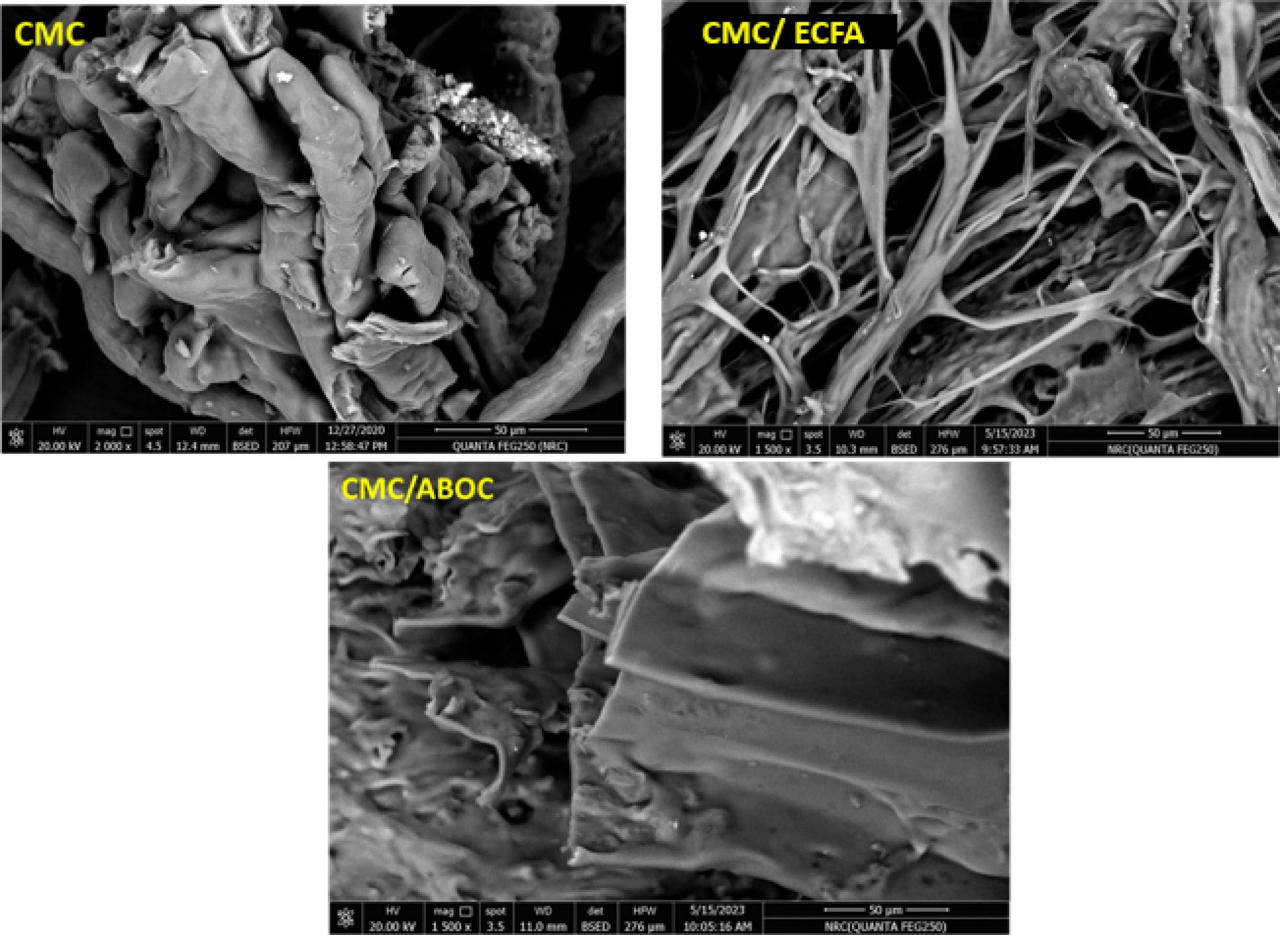
SEM of CMC and composites.

Figure 5 illustrates the surface morphology of CMC and CMC with heterocyclic compounds (ECFA, ABOC). The surface of CMC appears as collected fibers have a large thickness. While CMC/ECFA appears a small fibers, it seems as CMC has split through the interaction. Not only CMC/ECFA appears as a splitting fiber, but also CMC/ABOC looks like rows and layers put up to each other.

### Powder X-ray diffraction

**Fig. 6:**
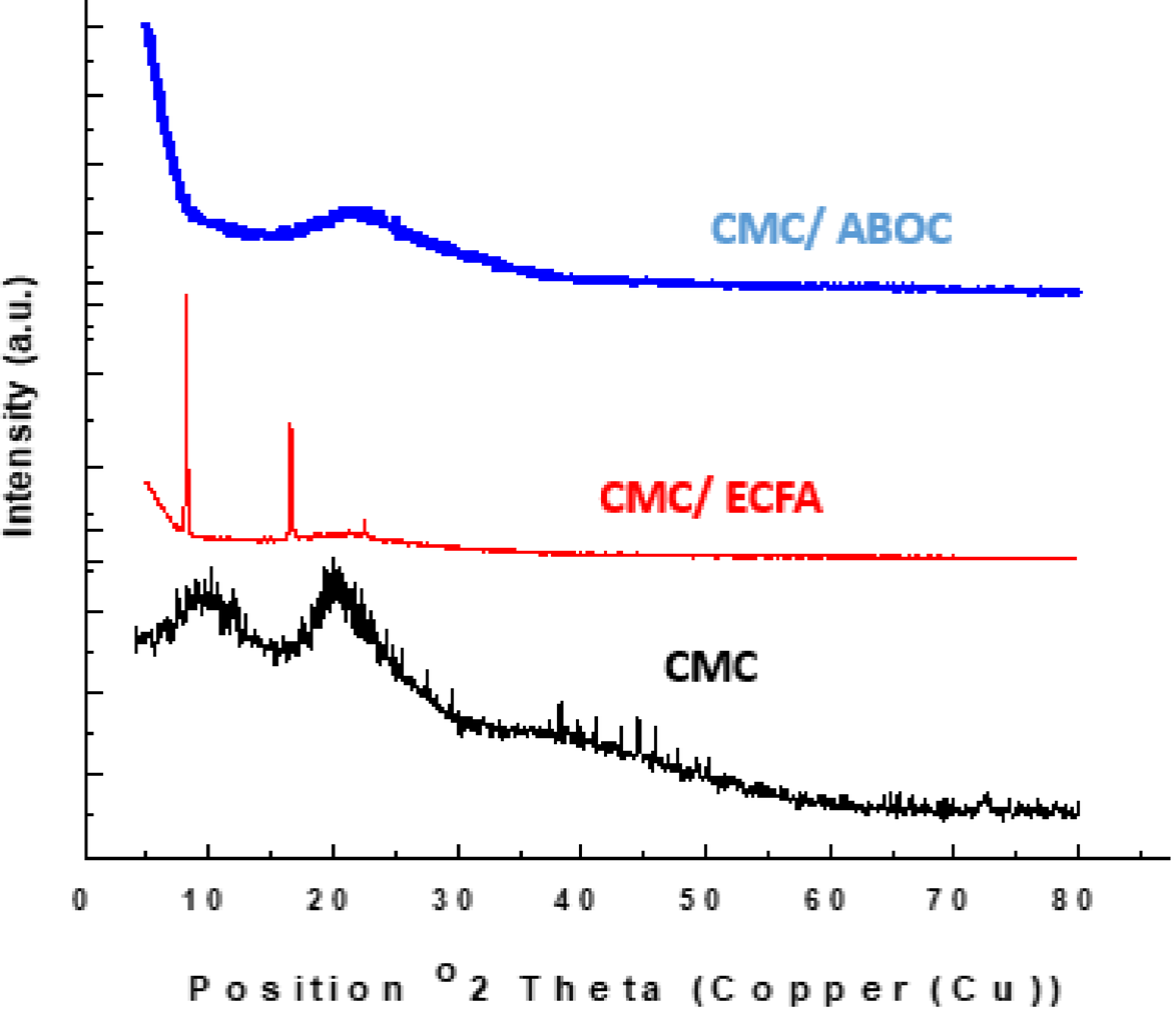
XRD of CMC and composites.

XRD analysis is indispensible method that used to identify the crystalline and amorphous region of prepared compounds. Figure 6 displays XRD pattern of CMC and CMC with heterocyclic compounds (ECFA, ABOC). The XRD pattern of CMC shows characteristic peaks of amorphous region at 2Ɵ=10^o^ and 20. While the XRD pattern of the CMC composite displays these characteristic peaks only slightly, revealing a change in crystallinity^34–36^.

## Evaluation of anticancer activity

### The cytotoxic effect of the synthesized compounds on different cell lines

The cytotoxic effect of the synthesized compounds was investigated with MTT assay on four cancer cell lines (HCT-116, MCF-7, PC3, and A549) and normal cell line RPE-1. First, we screened the new compounds at only one dose (100 µg/ml) for 48 h and the percentage of cytotoxicity was measured and compared to control untreated cells and positive control Doxorubicin. Results of this initial investigation showed that all compounds showed concentration dependent inhibitory effect against cell proliferation in all cell lines except MCF-7.

**Fig. 7:**
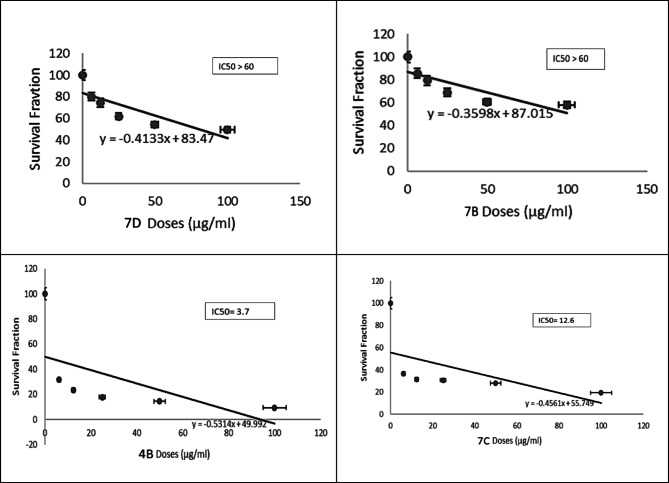
Effect of the synthesized compounds on HCT-116 Cell viability. The survival fraction of HCT-116 cells was assessed using the MTT assay after 48 h of treatment with varying concentrations of the synthesized compounds (6.25–100 µg/mL). Data are presented as mean ± SD from three independent experiments. *p < 0.01 compared to untreated cells.

**Fig. 8:**
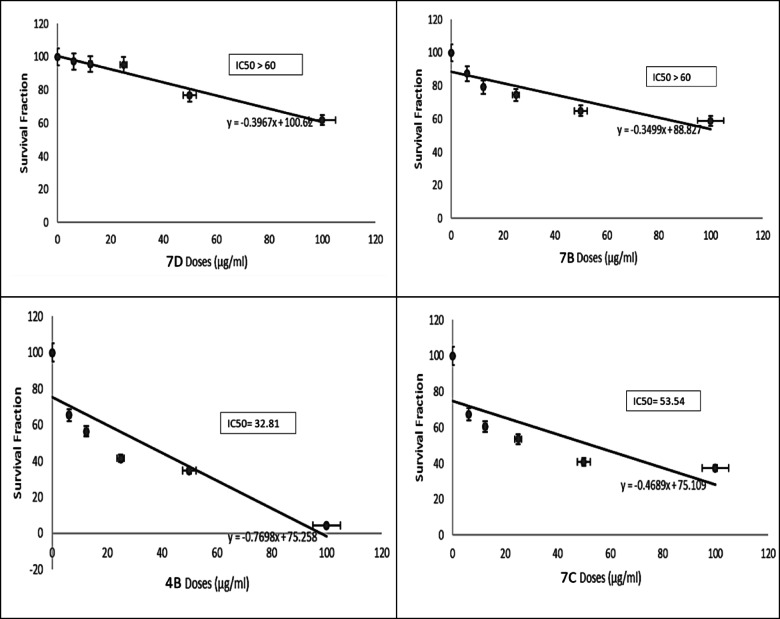
Effect of synthesized compounds on PC3 cells viability. Survival fraction evaluated using the MTT assay after 48 h of treatment with varying concentrations of compounds (6.25–100) µg/ml. Data are expressed as mean ± SD from three independent experiments. * p˂0.01 compared to untreated cells.

**Fig. 9:**
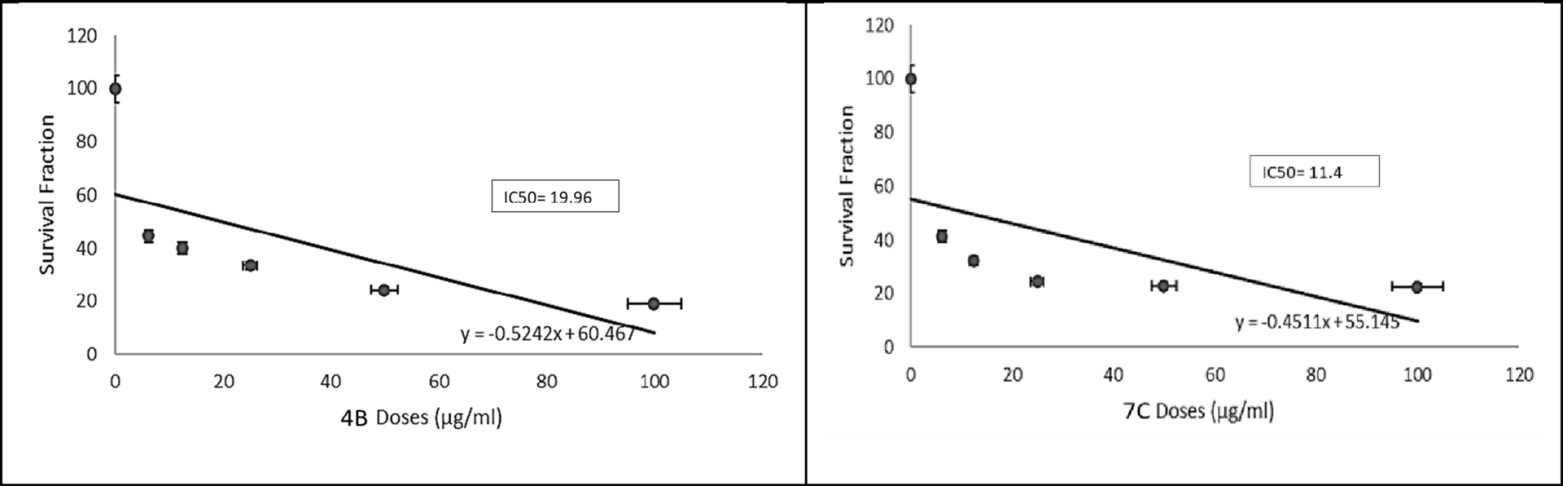
Effect of synthesized compounds 4b and 7c on A549 cells viability. Survival fraction was evaluated using the MTT assay after 48 h of treatment with varying concentrations of compounds 4b and 7c (6.25–100 µg/mL). Data are expressed as mean ± SD from three independent experiments. * p˂0.01 compared to untreated cells.

**Fig. 10:**
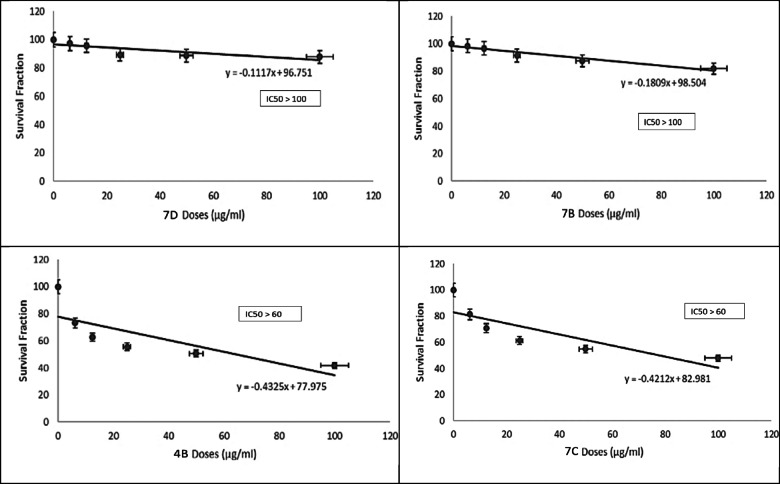
Effect of synthesized compounds on RPE-1 cells viability. The viability of RPE-1 cells was assessed using the MTT assay after 48 h of treatment with varying concentrations of the synthesized compounds (6.25–100 µg/mL). Data are expressed as mean ± SD from three independent experiments. *p < 0.01 compared to untreated cells.

After that, we used different concentrations of all CMC/ABOC compounds and studied their cytotoxic effect against the rest of cell lines including both normal and cancerous cells. According to results shown in Table 1, all compounds has cytotoxic effect against HCT-116, PC3 and A549 cells. The IC_50_ values were calculated for compounds which showed high cytotoxicity on different tested cell lines. As shown in (Figs. 7, 8, 9 and 10), compound CMC/ECFA and CMC/ABOC showed the highest significant cytotoxicity while HCT-116 and A549 cells were the most sensitive cells to our compounds. IC50 values of compound CMC/ECFA were 3.7, 32.81 and 19.96 µg/ml and for compound **7c** they were 12.6, 53.54 and 11.4 µg/ml against HCT-116, PC3 and A549 cells respectively. According to these results we can highlight the potent anticancer effect of compound **4b**,**7c** against HCT-116, PC3 and A549 cancer cells by recording the least IC50 values compared to untreated cells.

After investigating the significant anticancer effect of compound **4b**, **7c** we investigated their cytotoxicity against RPE-1 normal cells to determine their safety. It is known that the therapeutic safety of anticancer drugs is a crucial consideration for treating cancer patients. It is important factor for using these drugs clinically to avoid serious side effects. IC50 is commonly used to indicate the potency and sensitivity of an anticancer drug against cancer cells. The therapeutic safety of the drug can be evaluated by comparing the IC50 values and Hill slope of the anticancer drug in cancer cells relative to normal cells^37,38^. Fortunately, compounds **4b** and **7c** showed significant low toxicity against RPE-1 normal cells by recording very low IC50 values compared to studied cancer cells HCT-116, PC3 and A549.

According to our results, we can conclude that compounds **4b** and **7c** have high selectivity towards cancer cells and possess a strong safety margin for normal cells. This underscores their potential as potent and safe anticancer candidates for further investigation.

**Table 1:** Percentage of cytotoxicity of different concentrations of the synthesized compounds on cancer and normal cell lines.

	Cell line												
	HCT-116									A549			
Dose (µg/ml)	**7b**	**7c**	**7d**	**4b**		**7b**				**7c**		**7d**	**4b**
100	42.09	80.66*	50.36	90.56*		.				77.43*		.	80.93*
50	39.46	71.96*	45.53	85.50*		.				77.10*		.	75.73*
25	31.13	69.26*	38.09	82.12*		.				75.61*		.	66.61*
12.5	20.66	68.53*	25.46	76.53*		.				67.85*		.	60.04*
	**PC3**									**RPE-1**			
Dose (µg/ml)	**7b**	**7c**	**7d**	**4b**		**7b**				**7c**		**7d**	**4b**
100	40.98	62.56*	38.16	95.65*		18.06				52.28		12.14	58.13
50	35.06	59.17*	23.16	65.28*		12.54				45.25		11.29	49.43
25	25.40	46.43*	4.77	58.47*		8.61				38.66		10.76	44.43
12.5	20.74	39.51*	4.27	43.63*		3.27				29.23		4.21	37.33

* *p* < 0.01 when compared with untreated cells.

### The cytotoxic effect of the synthesized compounds on different cell lines

The cytotoxic effect of thesynthesized compounds was investigated with MTT assay on four cancer cell lines (HCT-116, MCF-7, PC3, and A549) and normal cell line RPE-1. First, we screened the new compounds at only one dose (100 µg/ml) for 48 h and the percentage of cytotoxicity was measured and compared to control untreated cells and positive control Doxorubicin. Results of this initial investigation showed that all compounds showed concentration dependent inhibitory effect against cell proliferation in all cell lines except MCF-7.

After that, we used different concentrations of all the targeted compounds and studied their cytotoxic effect against the rest of cell lines including both normal and cancerous cells. According to results shown at (Table 1), all compounds has cytotoxic effect against HCT-116, PC3 and A549 cells. The IC_50_ values were calculated for compounds which showed high cytotoxicity on different tested cell lines. As shown in (Figs. 6, 7 and 8), compound **4b** and **7c** showed the highest significant cytotoxicity while HCT-116 and A549 cells were the most sensitive cells to our compounds. IC50 values of compound **4b** were 3.7, 32.81 and 19.96 µg/ml and for compound **7c** they were 12.6, 53.54 and 11.4 µg/ml against HCT-116, PC3 and A549 cells respectively. According to these results we can highlight the potent anticancer effect of compound **4b**, **7c** against HCT-116, PC3 and A549 cancer cells by recording the least IC50 values compared to untreated cells.

After investigating the significant anticancer effect of compound **4b**, **7c** we investigated their cytotoxicity against RPE-1 normal cells to determine their safety. It is known that the therapeutic safety of anticancer drugs is one of the most important concerns of the physician treating the cancer patient. It is important factor for using these drugs clinically to avoid serious side effects. IC50 is usually used to represent the strength and sensitivity of an anticancer drug on cancer cells. The therapeutic safety of the anticancer drug can be assessed by comparing the IC50 and hillslope of anticancer drugs on cancer cells relative to normal cells^37,38^. Fortunately, compounds **4b** and **7c** showed significant low toxicity against RPE-1 normal cells by recording very low IC50 values compared to studied cancer cells HCT-116, PC3 and A549. According to our results, we can conclude that compounds **4b** and **7c** have high selectivity against cancer cells and high safety margins to normal cells, thereby highlighting their utilities as potent and safe anticancer drugs for further studies.

### Effect of the synthesized compounds on nitric oxide levels in LPS-stimulated RAW 264.7 macrophages

 The discovery of the potent activity of the synthesized compounds motivated us to further investigate the cellular mechanisms responsible for the potent cytotoxic effects observed against the cancer cells used in this study.

**Fig. 11:**
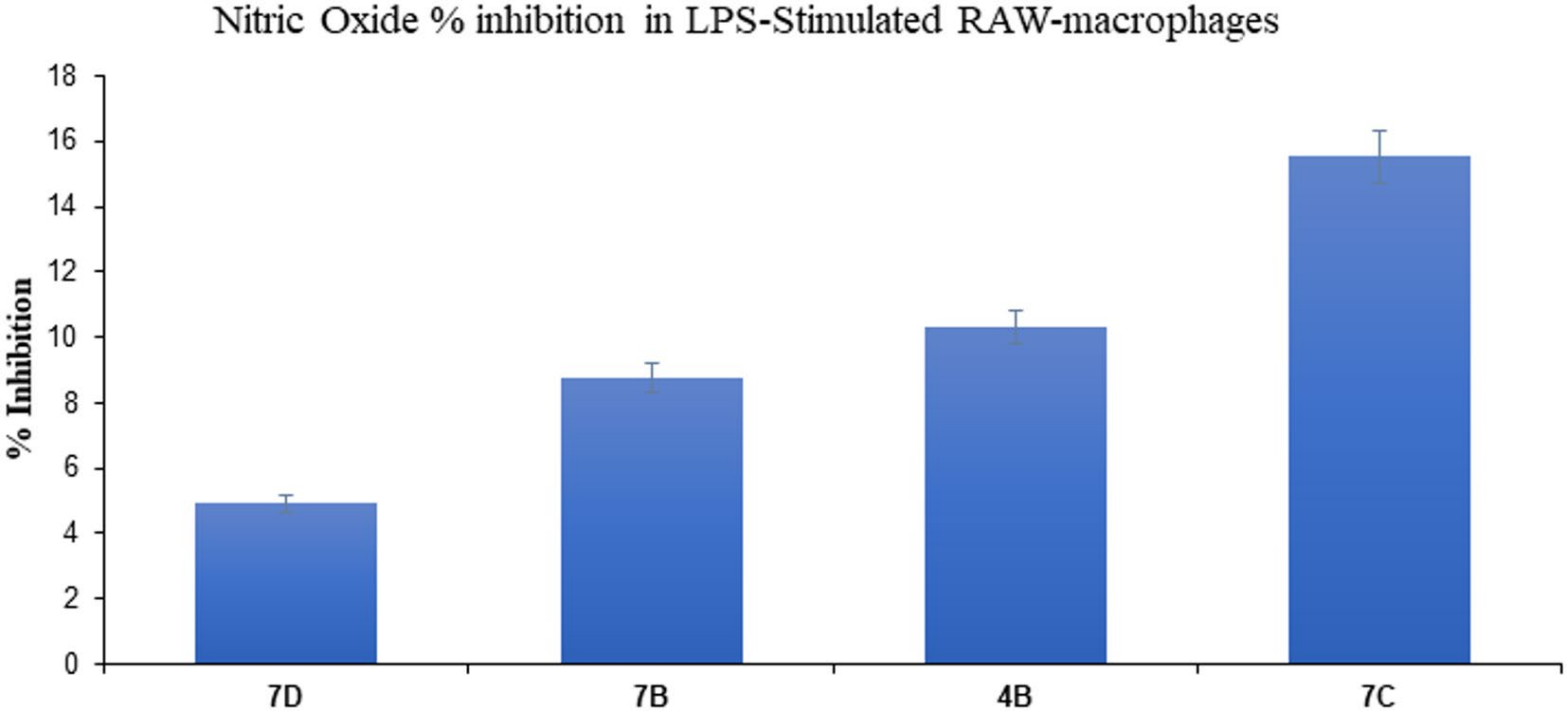
Effect of different compounds on the inhibition of nitric oxide production in LPS-stimulated RAW264.7 macrophages. RAW 264.7 macrophages were exposed to different compounds (100 µg/mL) along with LPS (1 µg/mL) or LPS alone for 24 h. Nitric oxide (NO) production was measured using the Griess reagent, and the results are expressed as mean ± SD (*n* = 3).

To achieve this goal, we studied the anti-inflammatory effect of these compounds. It is well documented that the inflammation is one of the cellular mechanisms involved in carcinogenesis^37‚39^.Accumulating evidence suggests that nitric oxide (NO) is a key mediator of inflammation. While NO plays a crucial role in various physiological functions, its excessive production, particularly in macrophages, can contribute to cytotoxicity, inflammation, and autoimmune disorders. The impact of different compounds on NO levels in LPS-stimulated RAW 264.7 cells was evaluated (Fig. 8). Cells were treated with various compounds in the presence of LPS or with LPS alone for 24 h. NO production was assessed by measuring nitrite levels in the culture medium utilizing the Griess reagent. The results showed that LPS alone significantly increased NO production compared to the control. However, pretreatment with the tested compounds did not inhibit NO release in LPS-stimulated RAW 264.7 cells (Fig. 11). These findings suggest that the anticancer effects of these compounds are independent of inflammation. Further studies are required to explore their precise mechanisms of action.

### Experimental

#### Synthesis of Ethyl 2-cyano-3-(5-methylfuran-2-yl)acrylate (4b)

A mixture of 3-methylfurfural (0.01 mol) and ethyl cyanoacetate (0.01 mol) was reacted at room temperature in ethanol in the presence of drops of piperidine. The precipitated solid was filtered off and re-crystallized from ethanol. Compound **4b** was afforded as a brown solid (90%); ^1^ H NMR (500 MHz, DMSO-d_6_): 1.24 (t, J = 7.5 Hz, 3 H, CH_3_), 2.40 (s, 3 H, CH_3_), 4.22–4.25 (m, 2 H, CH_2_), 6.52 (s, 1 H, CH), 7.43 (s, 1H, CH), 7.98 (s, 1H, CH). ^13^C NMR (125 MHz, DMSO-*d*_6_): 14.24, 62.58, 95.00, 99.98, 112.17, 116.21, 127.50, 139.07, 147.47, 161.40, 163.18. Elemental analysis calculated for C_11_H_11_NO_3_ (205.21): C, 64.38; H, 5.40; N, 6.83. Found: C, 64.35; H, 5.35; N, 6.80.

#### General procedure of synthesizing compounds 7

A mixture of 2-Cyano-*N*′-(aryl/heteroarylethylidene)aceto hydrazides (0.01 mol) and ethyl 2-cyano-3-(heteryl)acrylates was refluxed for three hours in methanol/acetic acid (1:2). The precipitated solid was filtered off and re-crystallized from methyl alcohol.

#### Synthesis of 6-amino-9-cyano-2,9a-dihydro-3-phenyl-8-(pyridin-3-yl)pyrido[2,1-*b*][1,3,4] oxadiazine-7-carboxylic acid (7b)

Compound **7b** was afforded as a yellow solid (74%); m.p.:308–310 °C, ^1^ H NMR (500 MHz, DMSO-d_6_): 4.46 (s, 2 H, CH_2_), 4.95 (s, 1 H, CH), 7.35–7.42 (m, 3 H, CH), 7.64 (s, 1 H, CH), 8.37–8.46 (m, 5 H, CH), 10.97 (br s, 3 H, COOH, NH_2_). ^13^C NMR (125 MHz, DMSO-*d*_6_): 35.00, 37.00, 42.10, 43.00, 83.15, 83.90, 117.13, 117.73, 124.19, 124.35, 135.46, 135.67, 136.15, 146.67, 149.21, 149.37, 149.83, 162.89, 163.01. Elemental analysis calculated for C_20_H_15_N_5_O_3_ (373.36): C, 64.34; H, 4.05; N, 18.76. Found: C, 64.32; H, 4.00; N, 18.70.

#### Synthesis of 6-amino-3-(4-bromophenyl)−9-cyano-2,9a-dihydro-8-(pyridin-3-yl)pyrido[2,1-*b*][1,3,4] oxadiazine-7-carboxylic acid (7c)

Compound **7c** was afforded as a yellow solid (83%), ^1^ H NMR (500 MHz, DMSO-d_6_): 4.47 (s, 2 H, CH_2_), 4.96 (s, 1 H, CH), 7.36–7.42 (m, 3 H, CH), 7.65 (s, 1 H, CH), 8.37–8.70 (m, 4 H, CH), 11.00 (br s, 3 H, COOH, NH_2_). ^13^C NMR (125 MHz, DMSO-*d*_6_): 35.00, 38.00, 41.10, 42.00, 83.13, 83.88, 117.13, 117.72, 124.22, 124.37, 135.73, 136.17, 136.38, 146.67, 149.02, 149.17, 149.33, 162.87, 163.00. Elemental analysis calculated for C_20_H_14_BrN_5_O_3_ (452.26): C, 53.11; H, 3.12; Br, 17.67; N, 15.49. Found: C, 53.09; H, 3.08; Br, 17.63; N, 15.44.

#### Synthesis of 6-amino-3-(3-aminophenyl)−9-cyano-2,9a-dihydro-8-(pyridin-3-yl)pyrido[2,1-*b*][1,3,4] oxadiazine-7-carboxylic acid (7d)

Compound **7d** was obtained as a yellow solid with an 81% yield and a melting point of 300–303 °C^29^.

#### Preparation of CMC composite

A homogeneous CMC solution was prepared by dissolving 0.5 g of CMC in 10 mL of distilled water under continuous stirring until fully dissolved. Then 0.15 g of heterocyclic compounds (AMOC, ABOC) was added with continues stirring to prevent agglomeration for 30 min. Afterward, the precursor was poured into in petri dish and frozen at − 20 °C, followed by a lyophilization process.

#### Characterizations

The characterization of the synthesized compounds was performed using various analytical techniques. FT-IR spectra were recorded in the range of 400–4000 cm − 1 using a Shimadzu 8400 S FT-IR spectrophotometer. The surface morphology was examined via scanning electron microscopy (SEM) using an FEI IN SPECTS electron microscope (Philips, Poland) in an environmental scanning mode without coating, along with a JEOL JEM-2100 electron microscope at 100k× magnification and an acceleration voltage of 120 kV.

X-ray diffraction (XRD) patterns were analyzed using a Diano X-ray diffractometer with a CuKα radiation source, operating at 45 kV. Additionally, a Philips X-ray diffractometer (PW 1930 generator, PW 1820 goniometer) was used, employing a CuK radiation source (λ = 0.15418\lambda = 0.15418λ = 0.15418 nm) within a diffraction angle range of 2θ from 10° to 80° in reflection mode.

Reaction progress was monitored using thin-layer chromatography (TLC) on pre-coated silica gel 60 F254​ aluminum plates, with visualization under UV light. The melting points of the compounds were determined utilized open capillary tubes on a Stuart SMP30 melting point apparatus and remained uncorrected.

The spectral analysis of the compounds was conducted at the Micro-analytical Labs of the National Research Centre in Cairo, Egypt. The ^1^H NMR spectrum was recorded using a Bruker Fourier 400 spectrometer at 400 MHz and 75 MHz, respectively, at 300 K.

## In-Vitro study

### Anticancer activity

#### Cell culture

The cytotoxic potential of the synthesized compounds was assessed against human carcinoma cell lines, including HCT-116 (colon), MCF-7 (breast), PC3 (prostate), and A549 (lung), along with the normal hTERT-RPE-1 (human immortalized epithelial) cell line. These cells were cultured in Dulbecco’s Modified Eagle’s Medium (DMEM) supplemented with 10% fetal bovine serum (FBS), penicillin (100 U/mL), and streptomycin (2 mg/mL). The cultures were maintained at 37 °C in a humidified atmosphere containing 5% CO₂ and 95% humidity. Cells were routinely passaged utilizing a 0.15% trypsin-versene solution.

The cell lines used in this study were generously provided by Professor Stig Linder from the Oncology and Pathology Department at the Karolinska Institute, Stockholm, Sweden, and were originally sourced from the American Type Culture Collection (ATCC).

### Cytotoxicity assessment on cancer monolayers

Cells were seeded in a 96-well plate at specific densities: 20,000 cells/well for HCT-116 and PC3 cell lines, 10,000 cells/well for A549 and MCF-7 cell lines, and 20,000 cells/well for the normal human immortalized epithelial cell line (hTERT-RPE1). After 24 h of incubation, the culture medium was aspirated and replaced with serum-free medium containing the test extracts (100 µg/mL). Cells were then treated for 48 h in triplicate.

Doxorubicin (100 µg/mL) served as the positive control, while 0.5% dimethyl sulfoxide (DMSO) was used as the negative control. Cell viability was determined using the MTT assay [3-(4,5-dimethylthiazol-2-yl)−2,5-diphenyltetrazolium bromide] following a previously established protocol^38^.

Cytotoxicity was calculated using the following equation:

% cytotoxicity = [1-(AVx/AVNC)]x100.

where *AVx* represents the average absorbance of the treated sample well, and *AVNC* corresponds to the average absorbance of the negative control well, measured at 595 nm with a reference wavelength of 690 nm.

### Determination of IC50 values

Compounds exhibiting cytotoxicity of ≥ 60% against various cancer cell lines were selected for dose-response studies at various concentrations. The final tested concentrations ranged from 100, 50, 25, 12.5, and 6.25 µg/mL down to 0.78 µg/mL, with all experiments performed in triplicate. IC50 values were determined by fitting the concentration-response curve to a non-linear regression model utilizing GraphPad Prism^®^ v6.0 (GraphPad Software Inc., San Diego, CA, USA).

## Anti‑inflammatory activity

### Cell culture (Seeding and Treatment)

The RAW 264.7 macrophage cell line was obtained from the American Type Culture Collection (ATCC). The cells were maintained in RPMI 1640 medium supplemented with 10% heat-inactivated fetal bovine serum and 1% penicillin-streptomycin. Prior to the experiment, the cells were subcultured twice and incubated in a humidified incubator at 37 °C with 5% CO₂.

### Procedure

All experiments were conducted in a clean environment using a biosafety class II laminar flow cabinet (Baker, SG403INT, Sanford, ME, USA). RAW 264.7 cells were suspended in RPMI medium and seeded at a density of 1 × 1051 \times 10^51 × 105 cells per well in 96-well plates. After 24 h of incubation, the cells were treated with test samples at concentrations of 100, 50, 25, and 12.5 µg/mL for 1 h. Following this, they were stimulated with 10 µg/mL lipopolysaccharide (LPS) for an additional 24 h.

After incubation, the supernatant was carefully transferred to a new 96-well plate for nitric oxide (NO) measurement. The remaining cells in the original plate were used for cell viability assessment utilizing the MTT assay. The test samples were prepared in RPMI medium after dissolving the stock solutions in DMSO.

Cell viability was determined based on the mitochondrial-dependent reduction of yellow MTT [3-(4,5-dimethylthiazol-2-yl)−2,5-diphenyl tetrazolium bromide] to purple formazan^40^. The percentage change in viability was calculated using the following formula:

((Reading of extract/Reading of negative control) −1) x 100^31^.

### Nitric oxide assay

The production of nitric oxide (NO) was assessed in the supernatants of cultured RAW 264.7 cells, following a previously described method with slight modifications^41^. Nitrite, a stable NO metabolite, was used as an indicator of NO production in the culture medium. After pre-incubating RAW 264.7 cells (1 × 1051 \times 10^51 × 105 cells/mL) with LPS (10 µg/mL) for 24 h, nitrite levels were measured using the Griess reagent. The reagent consisted of 1% sulfanilamide and 0.1% naphthyl ethylenediamine dihydrochloride in 2.5% phosphoric acid.

For the assay, 50 µL of cell culture supernatant was mixed with 50 µL of Griess reagent and incubated at room temperature for 15 min. The absorbance was then measured at 540 nm using a microplate reader. A fresh culture medium blank was included in each experiment. Nitrite concentration was determined using a sodium nitrite standard curve, as calculated by the following equation:

$${\text{Nitric Oxide inhibition (\% ) = }}\frac{{{\text{(control - Test)}}}}{{{\text{Control}}}}{{ \times 100}}$$

## Conclusion

Heterocycle-based compounds have been synthesized utilizing the 2-cyano-*N*′-(aryl/heteroarylethylidene)acetohydrazides and ethyl 2-cyano-3-(heteryl)acrylates. Based on the synthesized heterocycle-based compounds, the CMC-heterocycle composites were prepared. The structures of synthesized derivatives were confirmed using spectroscopic techniques such as NMR, fourier transform infrared spectroscopy (FTIR) as well as scanning electron microscopy (SEM). The investigations of anticancer effects of compounds on HCT-116, MCF-7, PC3 and A549 cancer cell lines were evaluated and their cytotoxicity against RPE-1 normal cells was estimated to determine their safety. Compounds **4b** and **7c** have high selectivity towards cancer cells and high safety margins to normal cells. The study demonstrated that the new heterocycle-based carboxymethyl cellulose conjugates are promising and could act as potential therapeutic agents.

## Supplementary Information

Below is the link to the electronic supplementary material.

Supplementary Material 1

## Data Availability

Data is provided within the manuscript.
