# Supplementary material for: Synthesis of heterocycle based carboxymethyl cellulose conjugates as novel anticancer agents targeting HCT116, MCF7, PC3 and A549 cells
Source: Sci Rep. 2025 Aug 9;15:29196. doi: 10.1038/s41598-025-14146-1 (PMC12335502; doi:10.1038/s41598-025-14146-1)
Supplement: Supplementary file 1 — Supplementary Material 1 [file 41598_2025_14146_MOESM1_ESM.docx]

**Supporting Information**

**Synthesis of** **heterocycle based carboxymethyl cellulose conjugates as novel anticancer agents targeting HCT116, MCF7, PC3 and A549 cells**

Reham A. Mohamed-Ezzat^1*^, Zeinab A. Elshahid^1^, Shaimaa A. Gouhar^2^, Sawsan Dacrory^3^

^1^Chemistry of Natural and Microbial Products Department, Pharmaceutical and Drug Industries Research Institute, National Research Centre, Cairo, Egypt.

^2^Medical Biochemistry Department, Medical Research and Clinical Studies Institute, National Research Centre, Cairo, Egypt,

^3^Cellulose and Paper Department, National Research Centre. Cairo, Egypt,

**Corresponding email:** [**reham_amgad_2010@yahoo.com**](mailto:reham_amgad_2010@yahoo.com) **;** [ra.mohamed-ezzat@nrc.sci.eg](mailto:ra.mohamed-ezzat@nrc.sci.eg)





**Compound 4b**


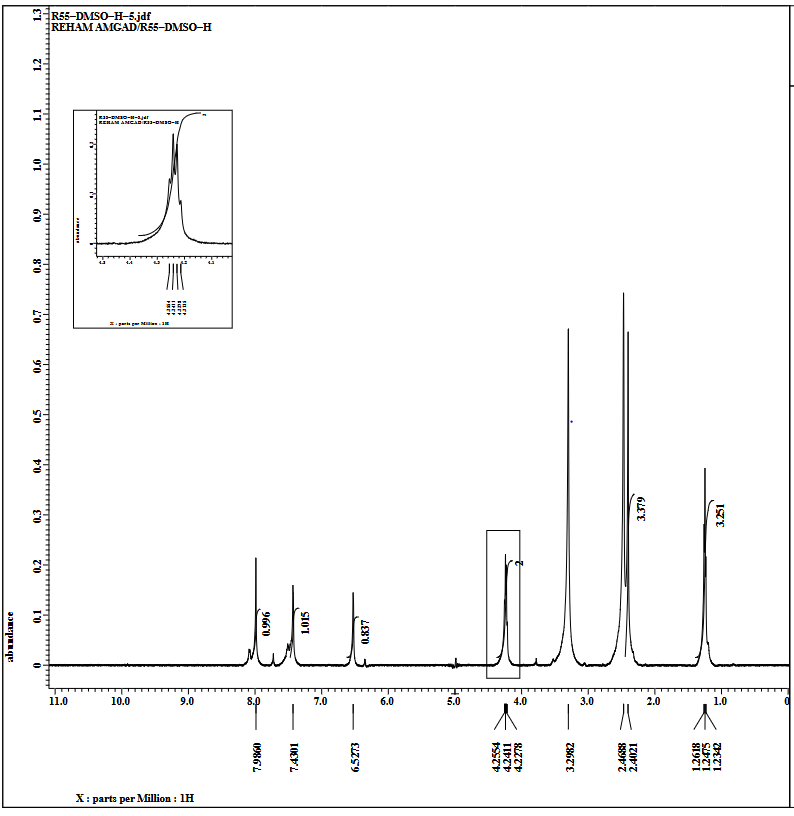


Figure S1: ^1^H -NMR spectrum of compound 4b


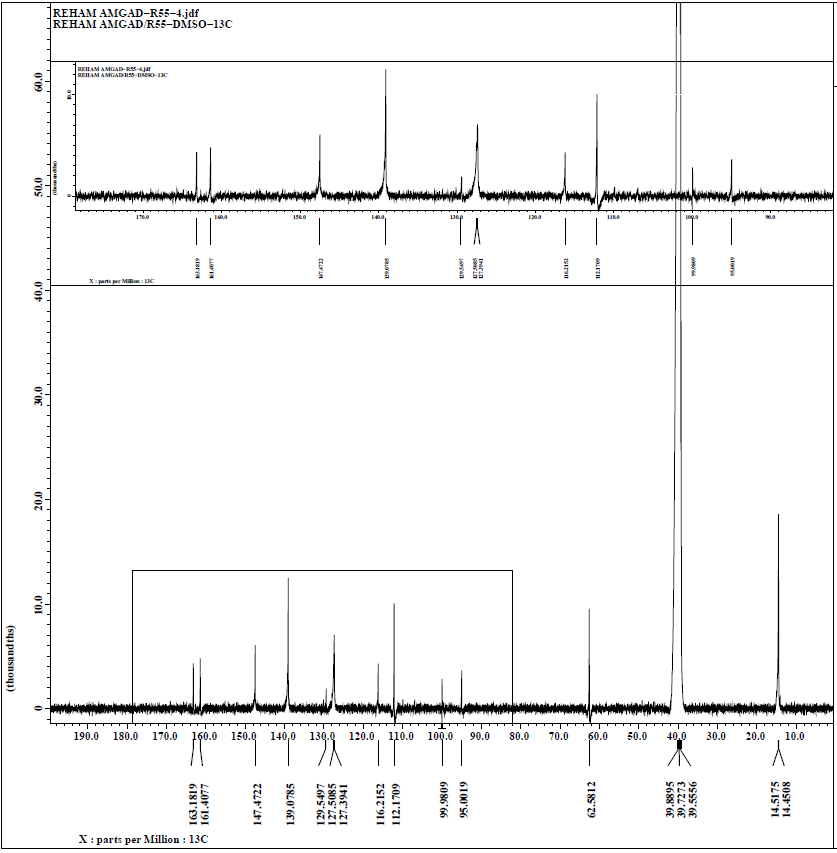


Figure S2: ^13^C-NMR spectrum of compound 4b





**Compound 7b**


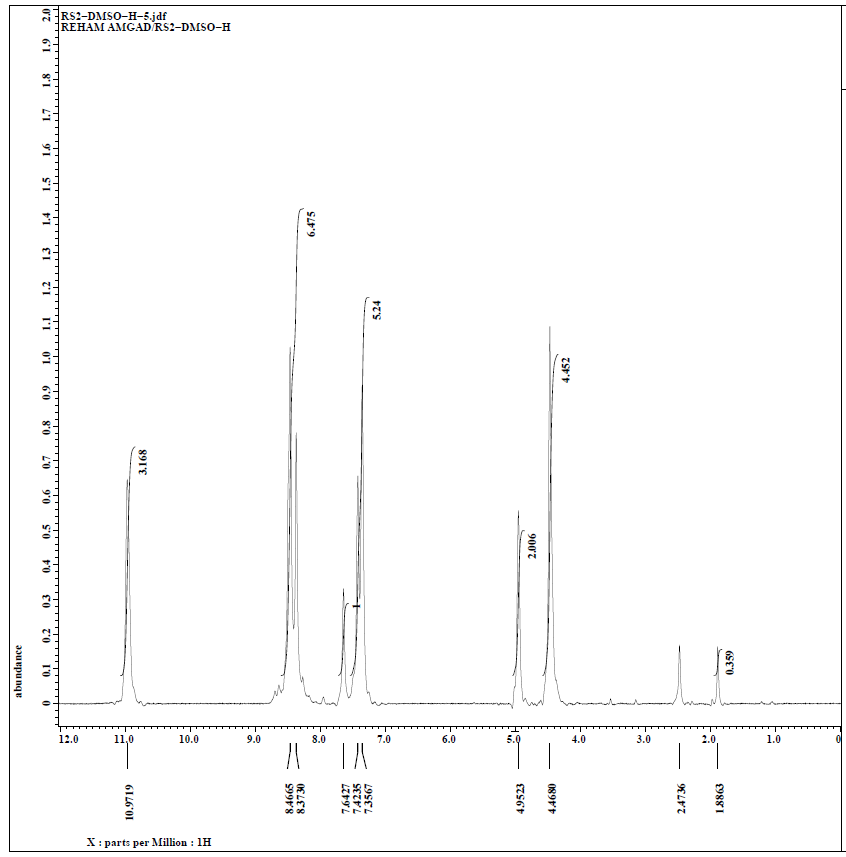


Figure S3: ^1^H-NMR spectrum of compound 7b


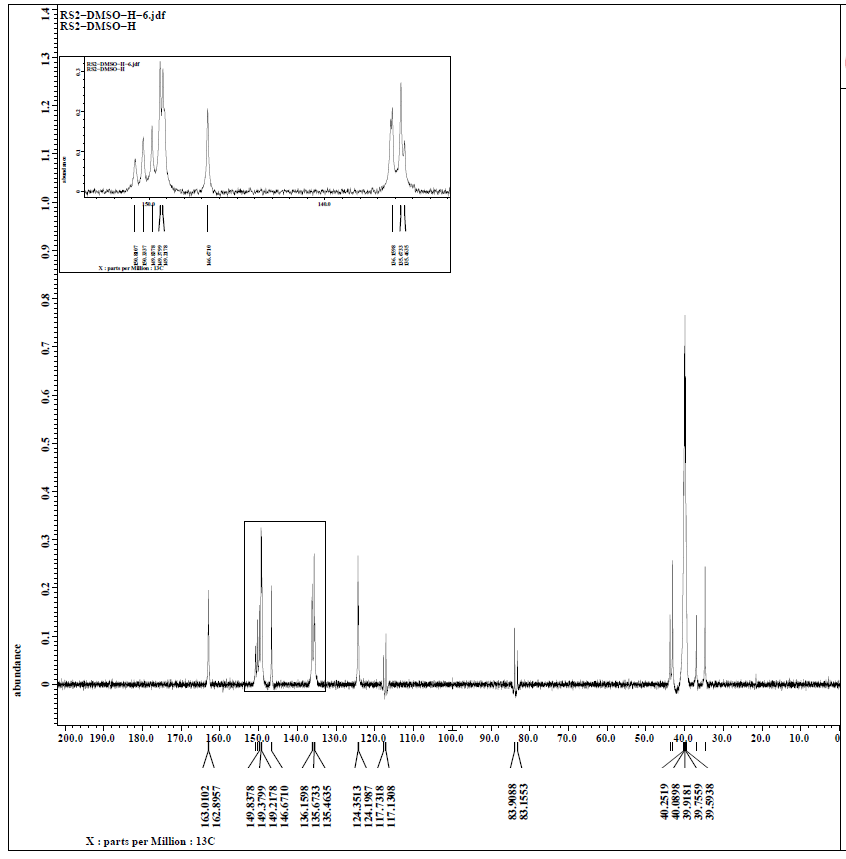


Figure S4: ^13^C-NMR spectrum of compound 7b





**Compound 7c**


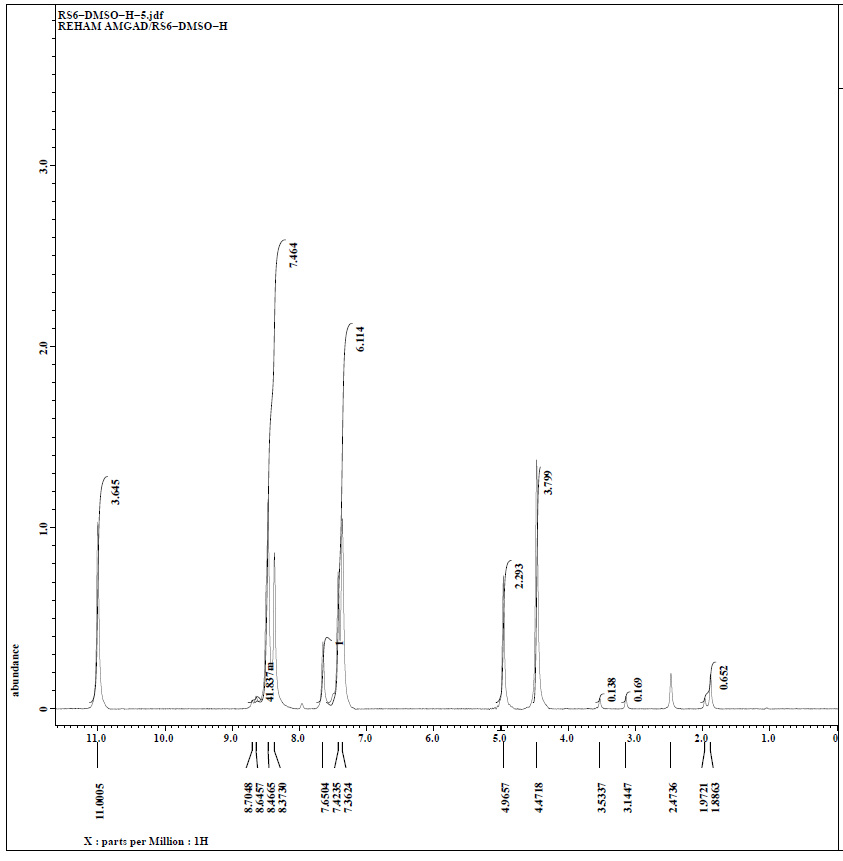


Figure S5: ^1^H-NMR spectrum of compound 7c


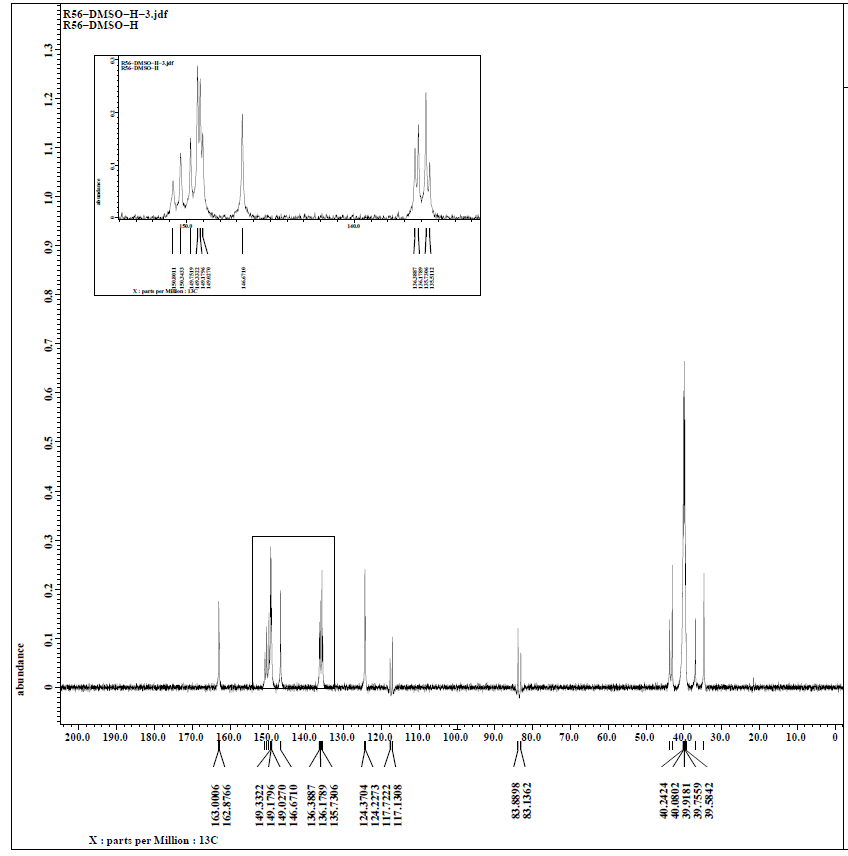


Figure S6: ^13^C -NMR spectrum of compound 7c
